# Efficacy of bariatric surgery in COVID-19 patients: An updated systematic review and meta-analysis

**DOI:** 10.1016/j.sipas.2022.100140

**Published:** 2022-10-28

**Authors:** Nimra Hasnain, Abdul Moeed, Eisha Waqar, Syed Ali Farhan, Fnu Amreek

**Affiliations:** a Department of Surgery, Ruth Pfau. Civil Hospital Karachi, Pakistan; b Dow Medical College, Dow University of Health Sciences, Karachi, Pakistan; c Department of Surgery, VCU Health, Richmond, VA, USA; d Department of Surgery, Yale New Haven Hospital, CT, USA

**Keywords:** Bariatric surgery, COVID-19, Weight loss

## Abstract

**Introduction:**

Obesity is linked with poor outcomes in patients with SARS-CoV-2 infection. In patients with BMI > 35 kg/m^2^, increased rates of hospital and subsequently ICU admissions have been noted. Bariatric surgery resulting in sustained weight loss is hypothesized to decrease the morbid outcomes associated with COVID. In this review, we update the evidence on the topic.

**Methods:**

An extensive literature search was conducted of electronic databases. Screening of the articles based on the eligibility criteria was followed by relevant data extraction. In addition to articles used in a previous meta-analysis, relevant databases were searched to filter for any new articles. Initially, two independent reviewers screened Pubmed and the Cochrane database followed by a thorough search of additional databases such as Google scholar and Medrxiv. Articles were first screened using title and abstract, followed by a full text read. Duplicates, meta-analysis, letter to the editors, and commentaries were excluded. No language restrictions were applied.

**Results:**

A total of nine articles with a population of 1,130,341 were entered into RevMan. Patients with bariatric surgery displayed significantly decreased hospitalization (OR: 0.52, 95% CI [0.45, 0.61]), were less likely to be admitted to the ICU (OR: 0.44, 95% CI [0.29, 0.67]), and had reduced overall mortality (OR: 0.42, 95% CI [0.25, 0.70]).

**Conclusion:**

Surgically induced weight loss is beneficial in reducing morbidity and mortality of COVID-19.

## Highlights

• Protective effects of bariatric surgery on COVID-19 patients are not well understood.
• Prior bariatric surgery was linked with decreased hospitalizations.
• Low overall mortality in the surgery group.
• Bariatric surgery was associated with less severe COVID-19 infection.

## Introduction

Obesity, previously associated with increased morbidity and mortality in respiratory viral infections [1], leads to worse clinical outcomes in patients with COVID-19. In a recent meta-analysis of patients infected with COVID-19, the risk of disease severity was 2.31 times higher in obese patients than non-obese patients [2]. Obesity has been further identified as a modifiable risk factor in COVID-19 patients for increased intensive care unit (ICU) admissions and invasive mechanical ventilation [3]. The pro-inflammatory effects of obesity can often supplement the pro-thrombotic effects of COVID-19, leading to detrimental disease processes such as deep vein thrombosis, myocardial infarction, stroke, and pulmonary embolism in COVID-19 infected patients [1].

Bariatric surgery is an established treatment modality for severe obesity and is associated with favorable long-term outcomes. It not only helps in achieving sustained weight loss but has also been shown to reduce co-morbidities associated with obesity such as hyperlipidemia, obstructive sleep apnea, hypertension, type-2 diabetes mellitus [4,5]. In light of the evidence in favor of the detrimental effects of obesity on COVID-19 infections, bariatric surgery has been researched as a mitigating factor and a treatment tool to enhance the quality of life in patients suffering from COVID-19.

Extensive evidence is now being generated indicating the protective effects of bariatric surgery on clinical outcomes related to severe COVID-19. A recent meta-analysis published by Aminian and Tu validated this rationale by concluding that prior bariatric surgery was associated with a lower rate of mortality and hospital admission in patients in obese patients infected with COVID-19 [6]. However, since COVID-19 is an ongoing pandemic, an increasing level of research is continuously being published on the subject. We aim to conduct an updated systematic review and literature search on the association between prior bariatric surgery and the clinical outcomes of COVID-19 infection. This will further help to promote the narrative of an ongoing double pandemic, i.e., COVID-19 and obesity, and subsequently alert public health authorities to resume and enhance access to bariatric surgery during the current pandemic.

## Methods

This meta-analysis is reported according to the Preferred Reporting Items for Systematic Review and Meta-Analysis (PRISMA) guidelines for updated meta-analysis [7] and follows the structure laid out by the Cochrane Collaboration [8].

### Data sources and search strategy

A detailed literature search of PubMed and Cochrane Central was conducted from its inception up until January 2022. Google scholar and Medrxiv.org were utilized as an additional search engines to screen any non-peer reviewed articles. The search strategy used in the electronic databases included keywords (“COVID-19 OR “coronavirus” OR “SARS-Cov-2”) and (“bariatric” OR “RYGB” OR “gastric bypass” OR “sleeve”). The complete search strategy is available in the supplementary material (S1). No restrictions on time, language, study design, and sample size were applied. Editorials and bibliographies of relevant review articles and unpublished databases were manually reviewed, ensuring none of the studies from white and grey literature were omitted.

### Study selection

Articles retrieved from the literature search were transferred to the Endnote Reference Library (Version X7.5; Clarivate Analytics, Philadelphia, Pennsylvania), where the duplicates were identified and removed. Two independent reviewers (N.H and A.M) used a two-phase blinded selection process. Articles were separately assessed according to their title and abstract details followed by a thorough full-text read. Selected articles were verified against the predefined inclusion criteria which were later matched and the duplicates were removed. In case of any conflict, a third reviewer (E.W) was consulted. Studies irrespective of language, containing full-text, and those dating to January 2022 were included. Meta-analyses and/or systematic reviews, letters to the editors (LTEs), and perspectives were excluded. Studies with patients with the following characteristics were included in the study: (a) COVID-19 positive and (b) with or without bariatric surgery. The primary end-points of the study were: mortality, hospital admission, and severe COVID-19 infection (ICU admission and mechanical ventilation combined).

### Data extraction and quality assessment

Following variables of interest were extracted from each study on a standard excel sheet: study population, study design, sample size, number of patients in each group (bariatric and non-bariatric), general patient characteristics of each group (age, gender, and race), comorbidities present amongst patients at baseline (hypertension, diabetes mellitus, hyperlipidemia, renal disease, and respiratory illness) and primary end-points including mortality, hospital admission, ICU admission, and mechanical ventilation. Two independent reviewers (E.W and A.M) conducted Quality Assessment to determine the risk of bias in each study. Newcastle-Ottawa scale [9] was employed for the pooled retrospective cohort studies. Studies were gauged on the selection, comparability, and outcomes of the representative cohorts. Those displaying most representativeness of the community, had least attrition loss, longer follow-up duration, and a standardized method to assess the outcome were attributed as having lower risk of bias. Any discrepancies found were resolved by consensus and discussion. (Table S1) Additionally, GRADE [10], an assessment tool to gauge the quality of systematic reviews/meta-analyses was employed. Studies included in the analysis are evaluated on the basis of risk of bias, inconsistency, indirectness, imprecision, and probability of a bias in publication. A detailed summary is provided in Table 2.

### Statistical analysis

This analysis was performed using Review Manager (RevMan) Version 5.4 Cochrane Collaboration. Generic invariance and the random-effects model were employed to derive odds ratios (ORs) and their corresponding 95% confidence intervals (CIs) for the pooled outcomes. Forest plots were used to represent each outcome. Higgins I^2^ statistics were utilized to assess the heterogeneity across the pooled studies. The value of I2 = 25–50% was considered mild, 50–75% moderate, and > 75% severe heterogeneity. A *p*-value < 0.05 was considered significant. Since there were less than 10 studies, the funnel plot could not be used to evaluate the publication bias.

## Results

### Study selection and characteristics

**Fig 1:**
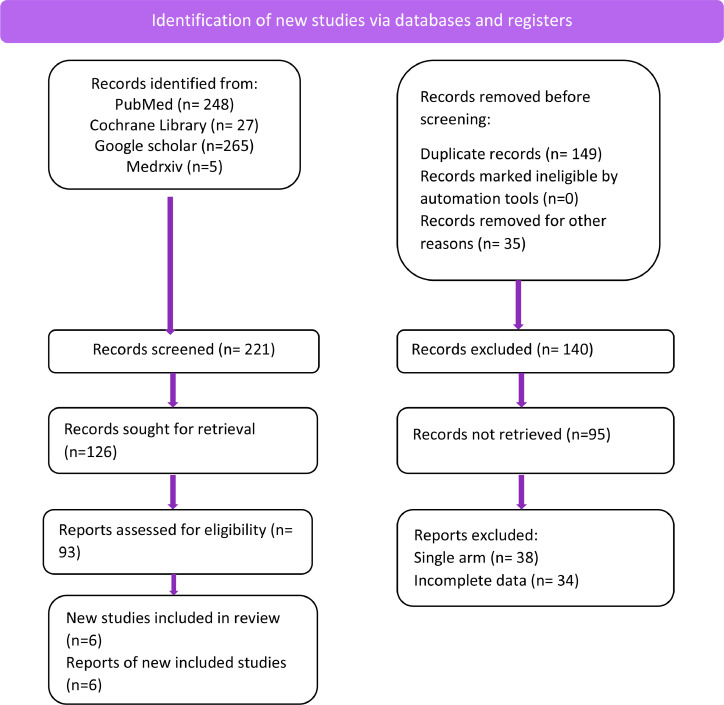
PRISMA model.

Our search of electronic databases yielded a total of 275 articles. After an initial screening of abstracts, 93 studies were assessed for detailed evaluation. Of these studies, a total of 9 studies [5,[10], [11], [12], [13], [14], [15], [16], [17]] satisfied our eligibility criteria and were included in the quantitative synthesis, as shown in Fig. 1. The remaining studies shortlisted for full-length assessment were excluded on being either single-arm (*n* = 38) or having incomplete data (*n* = 34). All nine studies included in the meta-analysis were retrospective cohorts with a total patient population of *n* = 1,130,341. Most studies were peer reviewed except for Iannelli et al. [12], were propensity matched cohorts, conducted analysis between 2020 and 2021, and contained patients tested positive for COVID through PCR. However, a few did not specify the screening method and included COVID susceptible cases as well [10,11]. Most were conducted in the US, however, a few were based on the Iranian [10] and French [11] databases. Except for Purdy et al. and Bramante et al. [5,13], studies specified the nature of the surgical technique, with majority including Roux-en-Y gastric bypass [11,12,[14], [15], [16], [17], [18]], sleeve gastrectomy [11,12,[14], [15], [16], [17], [18]], and gastric banding [12,14,17]. Among the included studies, females were the predominant sex in both bariatric and non-bariatric groups, with diabetes and hypertension being the common comorbidities. Characteristics of all included studies are present in Table. 1.

**Table 1:** Baseline characteristics of the patient population in each study; T*W= White, B= Black, O= Others, D=Declined, MBS-metabolic and bariatric surgery** Each % within a study corresponds to a different sample size.

**Study Name, Year**	**Sample size**		**Age, yrs**		**BMI, kg/m2**		**Gender, M, F %**		**Conclusion**
	MBS	Non-MBS	MBS	Non-MBS	MBS	Non-MBS	MBS	Non-MBS	
Aminian et al. (2020)	363		46.1 ± 12.7	49.8 ± 14.3	37.2 ± 7.1	46.7 ± 6.4	21.2,78.8	21.5, 78.5	Low risk of hospital and ICU admission in patients with obesity and MBS
Bramante et al. (2020)	373		56.1 IQR-43.5-65.3		35.3±8.2		47.1, 52.9		MBS was associated with a significant reduced risk of admission
Iannelli et al. (2020)	8285		49.8 ± 12.0	59.8 ±12.4	N/A	N/A	23.5, 76.5	5.8, 46.2	MBS associated with a lower risk of death and mechanical ventilation in patients with obesity and COVID-19
Hadi et al. (2021)	981,921		48.13 ± 11.88	48.62 ± 12.44	N/A	N/A	17.37, 82.32	17.37, 82.63	Prior MBS is affiliated with a decreased risk of poor outcomes of COVID-19
Jenkins et al. (2021)	620		51.7 ±12.6	52.1 ± 12.9	36.1 ± 8.3	41.4 ± 6.5	31, 69	31, 69	MBS significantly reduces the risk of ER admission, ICU stay, and mortality in patients with COVID-19
Blanchard et al. (2021)	2398		59.0 ± 10.8	59.8 ± 9.7	33.1 ± 5.4	33.0 ± 5.0	40, 60	41.4, 58.6	MBS in obese patients hospitalized for COVID-19 better prognostic outcomes than in non-MBS patients
Moradpour et al. (2021)	236		45.3 ± 11.3	45.1 ± 10.1	29.65 ± 6.2	45.08 ± 5.8	24.1,75.9	23.5, 76.5	MBS patients with COVID-19 had shorted hospitalization and ICU compared to non-MBS patients
Aminian et al. (2021)	11809		46.0 (38.0-55.0)	46.0 (34.0-56.0)	45.5 (40.9-51.4	45.3 (40.8-50.7)	21.4, 78.6	21.5,78.5	MBS was associated with reduced risk of hospitalization and severe disease in COVID-19 patients
Purdy et al. (2022)	124,699		N/A	N/A	N/A	N/A	27.6, 72.4	47.6, 52.4	MBS patients with obesity and COVID-19 had better outcomes compared to non-MBS patients

### Mortality (Fig. 2)

A random-effect analysis of eight studies reporting mortality was performed by pooling odds ratios (ORs) from dichotomous data. Compared with non-bariatric, the bariatric surgery group was significantly associated with an overall lower mortality (OR: 0.42, 95% CI [0.25, 0.70]; *p* < 0.0009; I^2^ = 73%).

**Fig. 2:**
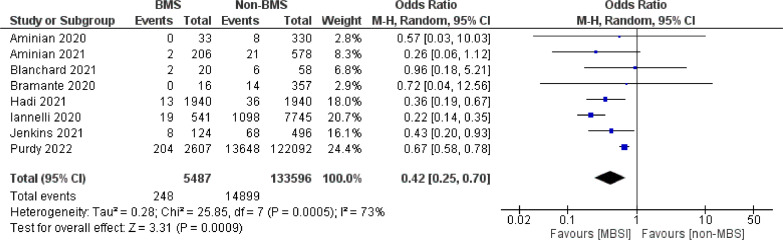
*Mortality in COVID-19 patients with bariatric surgery or without bariatric surgery***.** Blue squares and their corresponding lines are the point estimates and 95% confidence intervals per study. Black diamonds represent the pooled effect estimate.

### Severe COVID-19 infection (Fig. 3)

Eight studies assessing COVID-19 severity (mechanical ventilation and/or ICU admission) were pooled to derive ORs using a random-effects model. Patients undergone bariatric surgery in the past were significantly associated with developing a less severe COVID-19 infection (OR: 0.44, 95% CI [0.29, 0.67]; *p* < 0.0001; I^2^ = 83%).

**Fig. 3:**
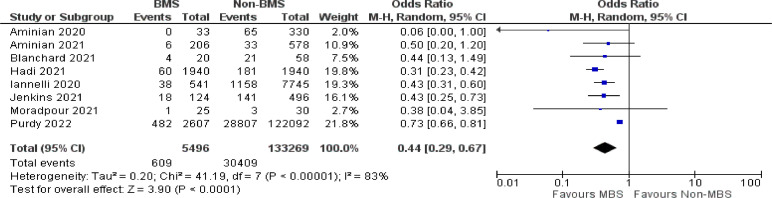
*Severe COVID-19 infection in patients with bariatric surgery or without bariatric surgery***.** Blue squares and their corresponding lines are the point estimates and 95% confidence intervals per study. Black diamonds represent the pooled effect estimate.

### Hospital admission (Fig. 4)

Pooling of six studies evaluating hospital admission in COVID-19 positive patients with or without bariatric surgery showed patients with bariatric surgery were less likely to be hospitalized (OR: 0.52, 95% CI [0.45, 0.61]; *p* < 0.00001; I^2^ = 0%).

**Fig. 4:**
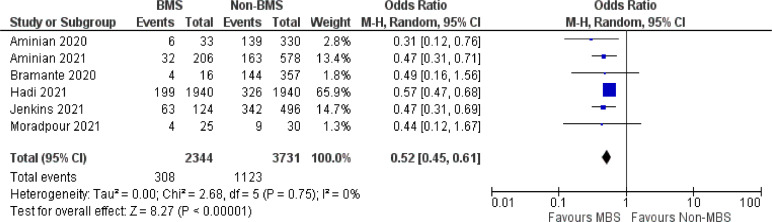
*Hospital admission in COVID-19 patients with bariatric surgery or without bariatric surgery***.** Blue squares and their corresponding lines are the point estimates and 95% confidence intervals per study. Black diamonds represent the pooled effect estimate.

### Sensitivity analysis

A leave-one-out sensitivity analysis was carried out to measure the degree of disproportionate effects produced by one single study. Purdy et al. [5] was identified as the study heavily influencing mortality and severe COVID-19 infection outcomes due to differences in the number and demographics of BMS and non-BMS groups. Despite the large population size, the methodology remains substandard due to the lack of individual patient data and multivariate analysis. Removal of Purdy et al. resulted in a decrease in heterogeneity from 73 to 0% in mortality and from 83 to 0% in severe COVID-19 infection. Both forest plots are present in *Supplementary Figs. 2 and 3*.

### Quality assessment

From the nine retrospective cohort studies included in this meta-analysis, one study, Moradpour et al. [11], was classified as of “Fair” quality, whereas the rest were of “Good” quality. Detailed quality assessment is included in the supplementary material (Table S2). GRADE rated all three outcomes as having moderate to critical importance and attributed the evidence as highly certain (Table 2).

**Table 2:** GRADE scoring system for the metaanalysis.

**Author(s):Nimra Hasnain,Abdul Moeed, Eisha Waqar, Syed Ali Farhan, Amreek KatariaQuestion:** Bariatric Surgery compared to Non-Bariatric Surgery in COVID-19 patients**Setting: -Bibliography:**. Bariatric Surgery vs Non-Bariatric Surgery in COVID-19 patients. Cochrane Database of Systematic Reviews [Year], Issue [Issue].												
**Certainty assessment**							**№ of patients**		**Effect**		**Certainty**	**Importance**
**№ of studies**	**Study design**	**Risk of bias**	**Inconsistency**	**Indirectness**	**Imprecision**	**Other considerations**	**Bariatric Surgery**	**Non-Bariatric Surgery**	**Relative (95% CI)**	**Absolute (95% CI)**		
**Severe COVID-19 Infection**												
8	observational studies	not serious	not serious	not serious	not serious	strong association all plausible residual confounding would suggest spurious effect, while no effect was observed	609/5496 (11.1%)	30409/133269 (22.8%)	**OR 0.44** (0.29 to 0.67)	**113 fewer per 1000** (from 149 fewer to 63 fewer)	⨁⨁⨁⨁ High	CRITICAL
**Hospital Admission**												
6	observational studies	not serious	not serious	not serious	not serious	all plausible residual confounding would reduce the demonstrated effect	308/2344 (13.1%)	1123/3731 (30.1%)	**OR 0.52** (0.45 to 0.61)	**118 fewer per 1000** (from 139 fewer to 93 fewer)	⨁⨁⨁◯ Moderate	IMPORTANT
**Mortality**												
8	observational studies	not serious	not serious	not serious	not serious	strong association all plausible residual confounding would reduce the demonstrated effect	248/5487 (4.5%)	14899/133596 (11.2%)	**OR 0.42** (0.25 to 0.70)	**61 fewer per 1000** (from 81 fewer to 31 fewer)	⨁⨁⨁⨁ High	CRITICAL

**CI:** confidence interval; **OR:** odds ratio.

## Discussion

This study is an updated meta-analysis that further confirms the protective function of bariatric surgery in patients infected with SARS-COV2, as stated by Aminian and Tu [6]. However, our meta-analysis consists of a larger population (*n* = 1,130,341 vs. 9022) and additionally evaluates the severity of infection. Patients admitted to the ICU and/or those who underwent mechanical ventilation were labeled with severe COVID-19 infection. Bariatric surgery was found to be unanimously beneficial; linked with decreased rates of hospitalization, less severe infection, and a more significant mortality benefit.

In patients infected with SARS-COV2, obesity has been attributed as the single most prognosis determining factor. According to a meta-analysis, individuals with obesity have an almost 50% higher risk of contracting SARS-COV2, with every patient getting hospitalized and every 3 in 4 at risk for ICU admission. Consequently, dismal outcomes such as the need for mechanical ventilation and ultimately death were observed in every 4 and 3 out of 6 individuals, respectively [19]. Moreover**,** intensive care patients, those having a BMI of > 35 kg/m^2^ are reported to be at a greater risk for disease progression [3]. Hypothetically**,** weight loss measures in general may ameliorate the severity of the disease and fasten recovery though surgical measures supersede pharmacological treatment.

Patients with bariatric surgery are 44% less likely to witness a severe event such as a mechanical event and/or ICU admission. The mortality rate in the surgical group is 42% less than in the nonsurgical group, which is higher (22%) than that reported by the previous meta-analysis [6]. Similarly, the rate of inpatient admissions in the surgical group was lower as compared to the previous meta-analysis [OR = 0.52, 95% CI (0.45–0.61) vs. 0.28, CI (0.12–0.65)]. The larger sample population in ours makes the protective benefit of bariatric surgery more explicit and lends validity to the hypothesized therapeutic effect of bariatric surgery.

The morbidity and mortality outcomes in COVID-19 patients seem to be dependent on two factors: (1) severity of the inflammatory response (2) primary lung function. Literature suggests that surgical weight loss measures assist in mitigating the damage posed by hyper-inflammatory states, such as that in COVID-19, as they help ‘calm’ the cytokine storm [20,21]. Additionally, in females, surgically induced weight loss is associated with a greater decrease in CD4 and CD3 T cells leading to a reduced inflammatory response [22]. However, these benefits are only witnessed following a significant period of sustained weight loss [23]. Additionally, morbidly obese patients have deranged pulmonary function tests, increased V/Q mismatch, lower ventilatory reserve, and are at a greater risk of developing right heart failure in cases of obstructive sleep apnea. Furthermore, prolonged ICU stay, acute lung events such as pneumonia and embolism, and intensive mechanical ventilation have been reported in such patients [24], [25], [26], [27]. This was similarly witnessed in patients with Middle Eastern respiratory syndrome (MERS-COV) and influenza. The former reported lower mortality rates in the surgical group, with the latter documenting lesser emergency admissions and shorter sick leaves [28], [29], [30]. Matos et al. found significant improvement in the pulmonary function tests following bariatric surgery, with respiratory efficiency becoming similar to that of controls after 6 months [31].

An improvement in the pre-existing co-morbidities indirectly impacts the progression of COVID-19. Other than sustained and prompt weight loss benefits of bariatric surgery, various obesity-related comorbidities are better controlled following surgical intervention. Bariatric surgery has been documented to benefit patients with increased HbA1C, hence, decreasing the morbidity and incidence of complication, followed by a substantial improvement in survival rates [32], [33], [34], [35], [36]. A meta-analysis by Buchwald et al. found that surgical measures reduced weight up to 60% and helped reduce metabolic parameters such as blood glucose total cholesterol, followed by improvement in hypertension and sleep apnea [37]. Therefore, current recommendations emphasize the protective role of bariatric/metabolic surgery and classify it as an emergency surgery in patients with ≥ 2 underlying chronic metabolic conditions [38]. These findings recommend the medical community to continue exploring safe surgical practices during the pandemics and establish better algorithms to prioritize those needing emergent care. Furthermore, additional studies stratifying the outcomes of bariatric surgery on the basis of demographics and metabolic profile must be conducted to get a more holistic view.

Like any other meta-analysis, this too has its inherent limitations. Firstly, as very few studies have reported their metabolic profile, subgroup analysis could not be performed. Secondly, although the majority of the data being propensity-matched eliminates the risk of heterogeneity I2, findings by Purdy et al. [5] render the data inhomogeneous (severe COVID-19 infection I2 = 83%, mortality = 73%). Other than that, the implicit bias of selective outcomes and data reporting cannot be gauged and eliminated as such. One example is the study by the University of Minnesota [13], which did not contribute to any heterogeneity, is not peer-reviewed, and hence, the findings may introduce bias. As seen in our analysis, females were more likely to undergo surgery than their male counterparts. However, multivariate regression revealed that the male gender presents with more severe disease, leading to increased morbid outcomes [39]. Due to the lack of individual outcomes in both genders, subgroup analysis could not be performed. Hence, the surgical benefit in COVID-19 patients could not be stratified based on gender. Although a few propensity-matched studies did not match BMI, race, and age, this did not affect the results as these factors were not included in our subgroup analysis. However, future meta-analyses may need to be wary of these confounders. Furthermore, the temporal relationship between surgery and contracting SARSCOV2 could not be evaluated due to the lack of data. In addition, assessment made by GRADE is rendered subjective, and recommendations made on the evidence provided should be dealt with caution. Although the findings of this meta-analysis lend credibility to the previous, future meta-analyses must evaluate for the missing endpoints.

## Conclusion

Bariatric surgery assists in alleviating the disease progression in patients with SARS-COV2. Decreased hospitalization, reduced ICU admissions, and lower mortality rates are positive outcomes in moderate to severe obesity patients. Future studies are needed to evaluate the endpoints points, highlight the confounders, and devise strategies for continual surgical access during pandemics.

## Funding

This research did not receive any specific grant from funding agencies in the public, commercial, or not-for-profit sectors.

## CRediT authorship contribution statement

**Nimra Hasnain:** Conceptualization, Visualization, Formal analysis, Validation. **Abdul Moeed:** Visualization, Formal analysis, Validation. **Eisha Waqar:** Writing – original draft. **Syed Ali Farhan:** Visualization, Writing – review & editing. **Fnu Amreek:** Writing – review & editing.

## Declaration of Competing Interest

The authors declare that they have no known competing financial interests or personal relationships that could have appeared to influence the work reported in this paper.

## Appendix Supplementary materials

Image, application 1

## References

[1] Kwok S., Adam S., Ho J.H., Iqbal Z., Turkington P., Razvi S. (2020). Obesity: a critical risk factor in the COVID-19 pandemic. Clin Obes.

[2] Yang J., Hu J., Zhu C. (2021). Obesity aggravates COVID-19: a systematic review and meta-analysis. J Med Virol.

[3] Simonnet A., Chetboun M., Poissy J., Raverdy V., Noulette J., Duhamel A. (2020). High prevalence of obesity in severe acute respiratory syndrome Coronavirus-2 (SARS-CoV-2) requiring invasive mechanical ventilation. Obesity.

[4] Arterburn D.E., Olsen M.K., Smith V.A., Livingston E.H., Van Scoyoc L., Yancy W.S. (2015). Association between bariatric surgery and long-term survival. JAMA.

[5] Purdy A.C., Hohmann S.F., Nguyen N.T. (2022). Outcomes of obese patients hospitalized with COVID-19: the impact of prior bariatric surgery. Surg Obes Relat Dis Off J Am Soc Bariatr Surg.

[6] Aminian A., Tu C. (2021). Association of bariatric surgery with clinical outcomes of SARS-CoV-2 infection: a systematic review and meta-analysis in the initial phase of COVID-19 pandemic. Obes Surg.

[7] Page M.J., McKenzie J.E., Bossuyt P.M., Boutron I., Hoffmann T.C., Mulrow C.D. (2021). The PRISMA 2020 statement: an updated guideline for reporting systematic reviews. BMJ.

[8] Cumpston M., Li T., Page M.J., Chandler J., Welch V.A., Higgins J.P. (2019). Updated guidance for trusted systematic reviews: a new edition of the Cochrane handbook for systematic reviews of interventions. Cochrane Database Syst Rev.

[9] Wells G., Shea B., O'Connell D., Peterson J., Welch V., Losos M.T.P.. The Newcastle-Ottawa Scale (NOS) for assessing the quality of nonrandomised studies in meta-analyses. 2013; Available from: http://www.ohri.ca/programs/clinical_epidemiology/oxford.asp.

[10] Schunemann H., Brozek J., Guyatt G., Oxman A. GRADE handbook [Internet]. [cited 2022 Oct 7]. Available from: https://gdt.gradepro.org/app/handbook/handbook.html.

[11] Moradpour G., Amini M., Moeinvaziri N., Hosseini S.V., Rajabi S., Clark C.C.T. (2022). Bariatric surgery, and COVID-19: what we have learned from the pandemic in Iran: a retrospective observational cohort study. Obes Surg.

[12] Iannelli A., Bouam S., Schneck A.S., Frey S., Zarca K., Gugenheim J. (2021). The impact of previous history of bariatric surgery on outcome of COVID-19. A nationwide medico-administrative French study. Obes Surg.

[13] Bramante C.T., Tignanelli C.J., Dutta N., Jones E., Tamaritz L., Clark J., et al. Non-alcoholic fatty liver disease (NAFLD) and risk of hospitalization for COVID-19. medRxiv. 2020 Jan 1;2020.09.01.20185850.

[14] Blanchard C., Perennec T., Smati S., Tramunt B., Guyomarch B., Bigot-Corbel E. (2021). History of bariatric surgery and COVID-19 19 outcomes in patients with type 2 diabetes: results from the CORONADO study. Obesity.

[15] Aminian A., Fathalizadeh A., Tu C., Butsch W.S., Pantalone K.M., Griebeler M.L. (2021). Association of prior metabolic and bariatric surgery with severity of coronavirus disease 2019 (COVID-19) in patients with obesity. Surg Obes Relat Dis.

[16] Hadi Y.B., Mann R., Sohail A.H., Graves M., Szoka N., Abunnaja S. (2022). Prior bariatric surgery is associated with a reduced risk of poor outcomes in COVID-19: propensity matched analysis of a large multi-institutional research network. Obes Surg.

[17] Jenkins M., Maranga G., Wood G.C., Petrilli C.M., Fielding G., Ren-Fielding C. (2021). Prior bariatric surgery in COVID-19-positive patients may be protective. Surg Obes Relat Dis Off J Am Soc Bariatr Surg.

[18] Aminian A., Tu C., Milinovich A., Wolski K.E., Kattan M.W., Nissen S.E. (2021). Association of weight loss achieved through metabolic surgery with risk and severity of COVID-19 infection. JAMA Surg.

[19] Popkin B.M., Du S., Green W.D., Beck M.A., Algaith T., Herbst C.H. (2020). Individuals with obesity and COVID-19: a global perspective on the epidemiology and biological relationships. Obes Rev Off J Int Assoc Study Obes.

[20] Zhang C., Zhang J., Liu W., Chen X., Liu Z., Zhou Z. (2019). Improvements in humoral immune function and glucolipid metabolism after laparoscopic sleeve gastrectomy in patients with obesity. Surg Obes Relat Dis.

[21] Bhatt D.L., Aminian A., Kashyap S.R., Kirwan J.P., Wolski K., Brethauer S.A. (2019). Cardiovascular biomarkers after metabolic surgery versus medical therapy for diabetes. J Am Coll Cardiol.

[22] Merhi Z.O., Durkin H.G., Feldman J., Macura J., Rodriguez C., Minkoff H. (2009). Effect of bariatric surgery on peripheral blood lymphocyte subsets in women. Surg Obes Relat Dis.

[23] Phillips C.L., Grayson B.E. (2020). The immune remodel: weight loss-mediated inflammatory changes to obesity. Exp Biol Med.

[24] Poirier P., Alpert M.A., Fleisher L.A., Thompson P.D., Sugerman H.J., Burke L.E. (2009). Cardiovascular evaluation and management of severely obese patients undergoing surgery: a science advisory from the American Heart Association. Circulation.

[25] Bercault N., Boulain T., Kuteifan K., Wolf M., Runge I., Fleury J.C. (2004). Obesity-related excess mortality rate in an adult intensive care unit: a risk-adjusted matched cohort study. Crit Care Med.

[26] Morgan O.W., Bramley A., Fowlkes A., Freedman D.S., Taylor T.H., Gargiullo P. (2010). Morbid obesity as a risk factor for hospitalization and death due to 2009 pandemic influenza A(H1N1) disease. PLoS One.

[27] Flegal K.M., Graubard B.I., Williamson D.F., Gail M.H. (2005). Excess deaths associated with underweight, overweight, and obesity. JAMA.

[28] Assiri A., Al-Tawfiq J.A., Al-Rabeeah A.A., Al-Rabiah F.A., Al-Hajjar S., Al-Barrak A. (2013). Epidemiological, demographic, and clinical characteristics of 47 cases of Middle East respiratory syndrome coronavirus disease from Saudi Arabia: a descriptive study. Lancet Infect Dis.

[29] Al-Tawfiq J.A., Assiri A., Memish Z.A. (2013). Middle East respiratory syndrome novel corona MERS-CoV infection. Epidemiology and outcome update. Saudi Med J.

[30] Valente M., Dalmonte G., Riccò M., Debs T., Gugenheim J., Iannelli A. (2021). Effects of bariatric surgery on influenza-like illness: a two-center cross-sectional study. Obes Surg.

[31] Matos C.M.P., Moraes K.S., França D.C., Tomich G.M., Farah M.W., Dias R.C. (2012). Changes in breathing pattern and thoracoabdominal motion after bariatric surgery: a longitudinal study. Respir Physiol Neurobiol.

[32] Aminian A., Zajichek A., Arterburn D.E., Wolski K.E., Brethauer S.A., Schauer P.R. (2019). Association of metabolic surgery with major adverse cardiovascular outcomes in patients with type 2 diabetes and obesity. JAMA.

[33] O'Brien R., Johnson E., Haneuse S., Coleman K.J., O'Connor P.J., Fisher D.P. (2018). Microvascular outcomes in patients with diabetes after bariatric surgery versus usual care: a matched cohort study. Ann Intern Med.

[34] Schauer P.R., Bhatt D.L., Kirwan J.P., Wolski K., Aminian A., Brethauer S.A. (2017). Bariatric surgery versus intensive medical therapy for diabetes - 5-year outcomes. N Engl J Med.

[35] Aminian A., Nissen S.E. (2020). Success (but Unfinished) story of metabolic surgery. Diabetes Care.

[36] Carlsson L.M.S., Sjöholm K., Jacobson P., Andersson-Assarsson J.C., Svensson P.A., Taube M. (2020). Life expectancy after bariatric surgery in the Swedish obese subjects study. N Engl J Med.

[37] Buchwald H., Avidor Y., Braunwald E., Jensen M.D., Pories W., Fahrbach K. (2004). Bariatric surgery: a systematic review and meta-analysis. JAMA.

[38] Rubino F., Cohen R.V., Mingrone G., le Roux C.W., Mechanick J.I., Arterburn D.E. (2020). Bariatric and metabolic surgery during and after the COVID-19 pandemic: DSS recommendations for management of surgical candidates and postoperative patients and prioritisation of access to surgery. Lancet Diabetes Endocrinol.

[39] Young M.T., Phelan M.J., Nguyen N.T. (2016). A decade analysis of trends and outcomes of male vs female patients who underwent bariatric surgery. J Am Coll Surg.

