# Supplementary material for: Efficacy of bariatric surgery in COVID-19 patients: An updated systematic review and meta-analysis
Source: Surg Pract Sci. 2022 Oct 28;11:100140. doi: 10.1016/j.sipas.2022.100140 (PMC9616481; doi:10.1016/j.sipas.2022.100140)
Supplement: Supplementary file 1 [file mmc1.docx]

**Online Supplementary**

Table S1: Detailed search strategy.

Figure S2: Sensitivity analysis of mortality.

Figure S3: Sensitivity analysis of severe COVID-19 infection.

Table S2: Newcastle-Ottawa Scale for cohort studies.

Table S3: Patient Characteristics.

| **PubMed** | ("COVID" OR "SARS-CoV-2" OR "coronavirus") AND ("bariatric" OR "RYGB" OR "gastric bypass" OR "sleeve") |
| --- | --- |
| **Cochrane Library** | ("COVID" OR "SARS-CoV-2" OR "coronavirus") AND ("bariatric" OR "RYGB" OR "gastric bypass" OR "sleeve") |

**Table S1: Detailed search strategy.**

**Figure S2: Sensitivity analysis of mortality.**

**
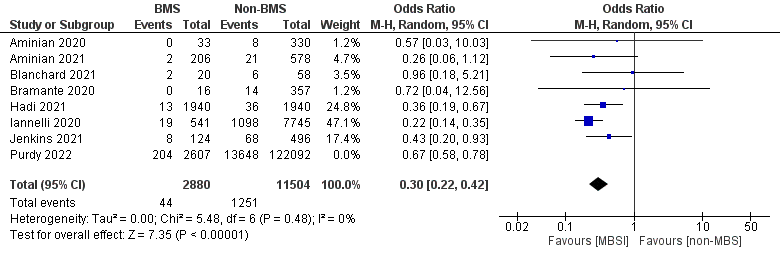
**

**Figure S3: Sensitivity analysis of severe COVID-19 infection.**

**
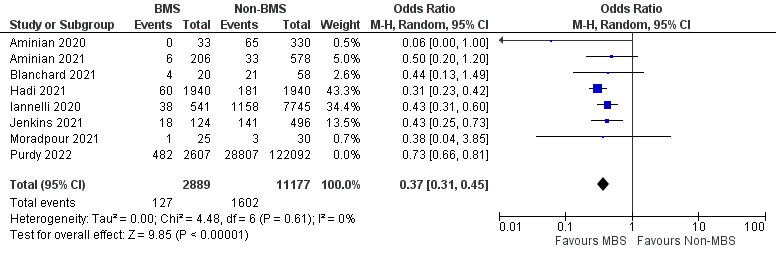
**

**Table S2: Newcastle-Ottawa Scale for cohort studies.**

|  |  |  |  |  |  |  |  |  |  |
| --- | --- | --- | --- | --- | --- | --- | --- | --- | --- |
| Study | **Representative nest of the exposed cohort** | **Selection of the non-exposed cohort** | **Ascertainment of exposure** | **Outcome not present at baseline** | **Comparability of the cohort** | **Assessment of outcome** | **Enough follow up duration** | **Adequate Follow-up** | **Total score** |
| Aminian et al, 2021 |  | * | * |  | ** | * | * | * | 7 |
| Bramante et al, 2020 |  | * | * |  | ** | * | * | * | 7 |
| Ianelli et al, 2020 |  |  | * |  | ** | * | * | * | 6 |
| Aminian et al JAMA, 2021 | * | * | * |  | ** | * | * | * | 8 |
| Hadi et al, 2021 | * | * | * |  | ** | * | * | * | 8 |
| Purdy et al, 2022 |  | * | * |  | * | * | * |  | 5 |
| Moradpour et al, 2021 |  | * | * |  | ** | * | * | * | 7 |
| Jenkins et al, 2021 |  | * | * |  | ** | * | * | * | 7 |
| Blanchard et al, 2021 | * | * | * |  | * | * | * | * | 7 |

| **Study Name, Year** | **Race, W, B, O /D** | | **Diabetes Mellitus, %** | | **Hypertension, %** | | | **COPD, %** | | **Heart Disease, %** | |
| --- | --- | --- | --- | --- | --- | --- | --- | --- | --- | --- | --- |
|  | MBS | Non-MBS | MBS | Non-MBS | MBS | | Non-MBS | MBS | Non-MBS | MBS | Non-MBS |
| Aminian et al 2020 | 48.5, 39.4, 12.1 | 46.7, 48.2, 5.1 | 6.1 | 34.2 | 36.0 | | 62.1 | 3.0 | 3.0 | 6.1 | 8.5 |
| Bramante et al 2020 | 46.2, 15.9, 37.8 | | 47.4 | | 70.8 | | | N/A | | N/A | |
| Iannelli et al 2020 | N/A | N/A | 12.2 | 36.8 | 20.2 | 41.6 | | 3.0 | 7.3 | 3.3 | 7.1 |
| Hadi et al 2021 | 26.55, 72.7 | 27.11, 71.34 | 39.28 | 38.92 | 68.92 | 69.23 | | 42.11 | 42.84 | 11.86 | 10.67 |
| Jenkins et al 2021 | 39, 30, 33 | 36, 29, 34 | 32 | 48 | 58 | 66 | | N/A | N/A | N/A | N/A |
| Blanchard et al 2021 | N/A | N/A | N/A | N/A | 70.0 | 80.7 | | 5.3 | 5.3 | 5.3 | 12.7 |
| Moradpour et al 2021 | N/A | N/A | 10.7 | 27.8 | 21.3 | 34.7 | | N/A | N/A | N/A | N/A |
| Aminian et al 2021 | N/A | N/A | 36.2 | 34.6 | 85.4 | 54.5 | | N/A | N/A | N/A | N/A |
| Purdy et al 2022 | 55.3, 25.6, 13.8 | 47.9, 23.9,  21.3 | 43.3 | 48.1 | 67.7 | 67.0 | | 30.2 | 25.8 | 18.7 | 17.3 |

**Table S3: Patient Characteristics.**
